# NapB Restores cytochrome *c* biosynthesis in bacterial *dsbD*-deficient mutants

**DOI:** 10.1038/s42003-022-03034-3

**Published:** 2022-01-21

**Authors:** Kailun Guo, Xue Feng, Weining Sun, Sirui Han, Shihua Wu, Haichun Gao

**Affiliations:** Institute of Microbiology and College of Life Sciences, Zhejiang University, Hangzhou, Zhejiang 310058 China

**Keywords:** Bacteriology, Water microbiology

## Abstract

Cytochromes *c* (cyts *c*), essential for respiration and photosynthesis in eukaryotes, confer bacteria respiratory versatility for survival and growth in natural environments. In bacteria having a cyt *c* maturation (CCM) system, DsbD is required to mediate electron transport from the cytoplasm to CcmG of the Ccm apparatus. Here with cyt *c*-rich *Shewanella oneidensis* as the research model, we identify NapB, a cyt *c* per se, that suppresses the CCM defect of a *dsbD* mutant during anaerobiosis, when NapB is produced at elevated levels, a result of activation by cAMP-Crp. Data are then presented to suggest that NapB reduces CcmG, leading to the suppression. We further show that NapB proteins capable of rescuing CCM in the *dsbD* mutant form a small distinct clade. The study sheds light on multifunctionality of cyts *c*, and more importantly, unravels a self-salvation strategy through which bacteria have evolved to better adjust to the natural world.

## Abstract (web summary)

The DsbD protein is normally required for cytochrome *c* maturation (Ccm) in bacteria. With cytochrome *c*-rich *Shewanella oneidensis* as the research model, NapB, the small subunit of the nitrate reductase which is a cytochrome *c* per se, was found to suppress the Ccm defect resulting from DsbD loss under anaerobic conditions.

## Introduction

Cytochromes *c* (cyts *c*), ubiquitous heme-containing proteins present in all domains of life, are primarily involved in energy transduction processes as electron carriers in respiration and photosynthesis^1^. Unlike other hemoproteins, these proteins are characterized by that heme *b* is covalently attached to heme binding motifs (HBM, typically CX_2_CH), a posttranslational modification process called cyt *c* maturation (CCM, also called cyt *c* biosynthesis)^1^. Among the systems catalyzing the process, Ccm systems, present in diverse Gram-negative bacteria and archaea, as well as in plant and protozoan mitochondria, consisting of either eight or nine proteins, such as CcmABCDEFGH in *Escherichia coli* and CcmABCDEFGHI in *Rhodobacter capsulatus* and *Shewanella*, are undoubtedly the most complex^1–4^.

**Fig. 1:**
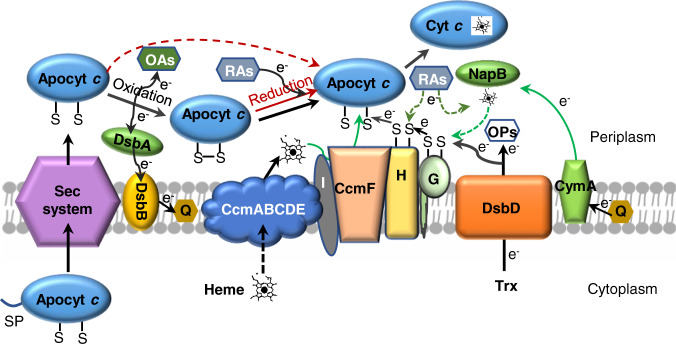
Proposed model for CCM in *S. oneidensis*. The model illustrates oxidation and reduction of apocyts *c*. In *S. oneidensis*, a large majority of apocyts *c* can be oxidized by DsbA proteins and by oxidative agents (OAs). These oxidized apocyts *c* rely on reducing equivalents from DsbD via CcmG and CcmH for their reduction such that covalent heme ligation could occur on CcmF. A residual quantity of reduced apocyts *c* (red dash line) can be matured by the CCM system without needing electrons from DsbD. In the absence of DsbD, NapB can donate electrons to CcmG for reduction of the oxidized apocyts *c*. Reducing agents (RAs) may prevent oxidation of apocyts *c* (red dash line) or reduce oxidized apocyts *c* (red solid line). RAs may also donate electrons to CcmH and/or NapB (dark green dash lines). Q quinol species, SP signal peptide, OPs other processes.

After synthesis in the cytoplasm, apocyts *c* are exported into the periplasmic compartment via the classical Sec protein secretion apparatus, where the two cysteines of any HBM are promptly oxidized to form a disulfide bond^5^ (Fig. 1). This process, catalyzed by the DsbA/DsbB oxidative folding system as well as other accessory proteins and/or small molecule oxidants, has to be promptly carried out as unoxidized apocyts *c* are degraded rapidly^6–9^. In the meantime, heme *b* molecules also need to be transported into the periplasm from the cytoplasm, presumably by the CcmABCDE function module^4,10^. For heme ligation to HBM, the disulfide bonds within apocyts *c* have to be converted back to the reduced state, a process that relies on DsbD^11^. DsbD is an inner-membrane protein transferring electrons from the cytoplasmic thioredoxin system not only to the Ccm system but also to various redox pathways present in the cell envelope^12^. Electrons from DsbD pass through CcmG and then CcmH to lyase CcmF, where reduction of oxidized apocyts *c* and thioether bond formation between the Cys residues within HBM and heme occur^1,5^. While CcmG is proposed to be periplasmic thioredoxin catalyzing the reduction of apocyt *c*, CcmH is closely associated with CcmF also likely serving as apocyt *c* chaperon^13,14^.

In addition to Ccm components, DsbD and its functional orthologs are well recognized to be essential for CCM when DsbA/DsbB is functioning^4,12^. However, a small quantity of cyts *c* can mature independent of DsbD if DsbA/DsbB is absent^7,15,16^. This DsbD-independent CCM can be promoted by small reducing agents, such as cysteine, reduced glutathione (GSH), and so on^7^.

*Shewanella*, a group of ubiquitous γ-proteobacteria, exhibit a remarkable versatility in respiration, allowing the use of an array of diverse electron acceptors (EAs), including oxygen, fumarate, nitrate, nitrite, thiosulfate, trimethylamine *N*-oxide (TMAO), dimethylsulfoxide, Fe(III), Mn(III) and (IV), Cr(VI), U(VI), and so forth^17,18^. This feature, endowing the bacteria great potential in bioremediation of heavy metals and energy generation via microbial fuel cells, is a long-time evolution consequence of adaptation to redox-stratified environments where these bacteria primarily reside^17,19^. A large repertoire of cyts *c* (up to 42 in the genus representative *S. oneidensis*) are not only the basis of the respiratory versatility but also confer on *Shewanella* strains (colony and cell-pellet) a red-brown color, a phenotypic signature that greatly facilitates studies of CCM because the abundance of cyts *c* correlates well with the color intensity^20–22^.

For anaerobic respiration, cyts *c* are needed to transport electrons from the quinol pool to terminal respiratory reductases, many of which per se are cyts *c*^23,24^, contrasting that oxygen respiration catalyzed by cyt *bd* oxidase utilizes electrons directly from the quinol pool^19^. Thus, in *Shewanella*, as best illustrated in *S. oneidensis*, respiration of non-oxygen EAs is dependent on cyts *c* whereas oxygen can be respired in their absence^19,25^. Not surprisingly, *S. oneidensis* cells grown on non-oxygen EAs possess increased cyt *c* abundance compared to those grown on oxygen^21,26^. The increase is largely achieved through cAMP, which serves as the activating cofactor for Crp (cAMP receptor protein), the predominant transcriptional regulator for respiration in *Shewanella*^27,28^. In anaerobically growing cells intracellular concentrations of cAMP are substantially higher than those in cells respiring oxygen, leading to an enhanced abundance of cyts *c*^19,25,28,29^.

Compared to the regulation of cyt *c* abundance, it is conceivable that the mechanisms ensuring CCM in natively living *Shewanella* cells are more critical given that non-oxygen EAs dominate in the stratified niches. *S. oneidensis* possesses a highly conserved Ccm system, but its components are encoded by three operons, *ccmABCDE* (heme transport), *ccmI*, and *ccmFGH* (heme-apocyt *c* ligation) in the *ccm* locus, in contrast to a single operon for the *ccm* genes present in most other γ-proteobacteria^2,22^. This functional modularity design is presumably a means to increase resilience to disruptive mutations. In parallel, *S. oneidensis* possesses three DsbAs and two DsbBs for oxidation of apocyts *c*, possibly to minimize degradation of apocyts *c* because these proteins in the unoxidized form are unstable and highly susceptible to periplasmic proteases^6–9^. In contrast, there is only one DsbD encoded in the genome, posing an unreasonable threat to the survival and growth of cells when non-oxygen EAs are present.

In this study, we continued our effort to unravel mechanisms through which *S. oneidensis* adapts to redox-stratified environments. By accident, we observed that an *S. oneidensis dsbD* null mutant can grow and produce cyts *c* under anaerobic conditions. By using random mutagenesis, we identified that cyt *c* NapB underpins suppression of the CCM deficiency resulting from the DsbD loss. The suppression can be achieved only under anaerobic conditions because NapB is considerably produced due to the activation of cAMP-Crp. Further investigations revealed that NapB functions to reduce CcmG as DsbD does, thereby leading to the reduction of oxidized apocyt *c*. This activity is only observed from a small portion of NapB proteins, which are classified into β-type characterized by that they lack specific electron donor NapC. Seemingly, β-type NapBs function as a promiscuous electron mediator whose reduction and oxidation can be coupled to diverse redox agents.

## Results

### *S. oneidensis* strain lacking DsbD can grow both aerobically and anaerobically

**Fig. 2:**
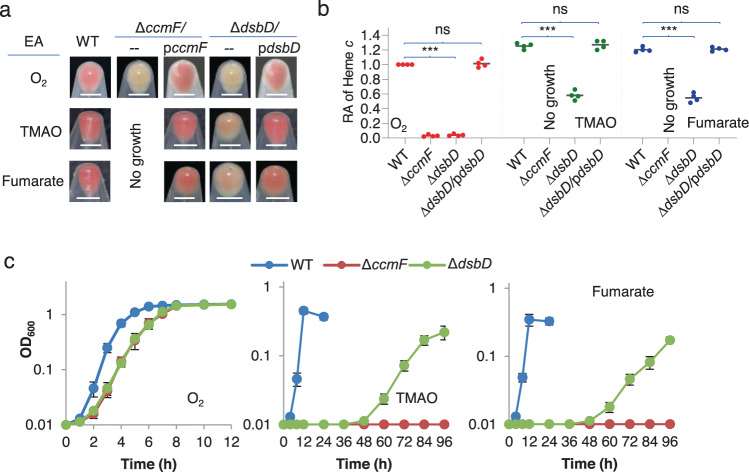
*S. oneidensis dsbD* mutants can grow both aerobically and anaerobically. **a** The cell color phenotypes of indicated strains. Shown were cell-pellets of indicated strains grown on one of EAs, O_2_, TMAO, and fumarate, to the early stationary phase. Strains include WT (the wild-type), ∆*dsbD* and ∆*ccmF* carrying the empty vector (--) and respective complementary vectors (p*dsdD* and p*ccmF*). Throughout this study, all strains without expressing a plasmid-borne gene carried empty vectors unless otherwise noted. Scale bars shown in all subpanels, 5 mm. **b** Heme *c* levels. Samples used in (**a**) were lysed for quantification of heme *c*. After quantification (Supplementary Fig. 2a), the data were first adjusted according to protein levels of samples, and then the averaged levels of the mutants were normalized to that in the wild-type, which was set to 1, giving to relative abundance (RA). Asterisks indicate statistically significant difference of the values compared (*n* = 4; ns, not significant, *p* > 0.05; *P < 0.05; **P < 0.01; ***P < 0.001). **c** Growth of indicated strains on EAs specified. Growth data of complementary strains were given in Supplementary Fig. 2b. All experiments were performed four times, and representative data as in (**a**), all data as in (**b**), or average ± SD (error bar) as in (**c**) were presented.

Given that cyts *c* are responsible for the well-known red-brown color of *S. oneidensis* colonies and cell-pellets and the color intensity is well correlated to the overall cyt *c* content^7,22^, we took advantage of this feature for rapid assessment of the cyt *c* abundance in all strains used in this study (Fig. 2a, b and Supplementary Figs. 1,  2a). In line with the essentiality of DsbD in bacterial CCM, the *S. oneidensis dsbD* null mutation abolishes CCM in cells grown aerobically, and consistently cell-pellets lose the characteristic color^1,7,30^ (Fig. 2a, b and Supplementary Fig. 1).

By accident, we found that the ∆*dsbD* strain was able to grow on TMAO and fumarate, albeit with an extremely long lag phase and at lowered growing rates (Fig. 2c). Not surprisingly, these cells were able to produce cyts *c*, to ~ 40% relative to the wild-type (Fig. 2a, b). In contrast, a cyt *c*-deficient strain (∆*ccmF*) devoid of an essential component of the Ccm system (heme lyase CcmF), was unable to grow anaerobically although it exhibited growth, cell-pellet color, and cyt *c* content indistinguishably from the ∆*dsbD* strain under aerobic conditions^3,22^ (Fig. 2a–c). As genetic complementation for both mutants was successful (Supplementary Fig. 2b), these data manifest that CCM can occur independently of DsbD in *S. oneidensis* cells living in anaerobic environments.

### cAMP-Crp is involved in anaerobic growth of ∆*dsbD*

**Fig. 3:**
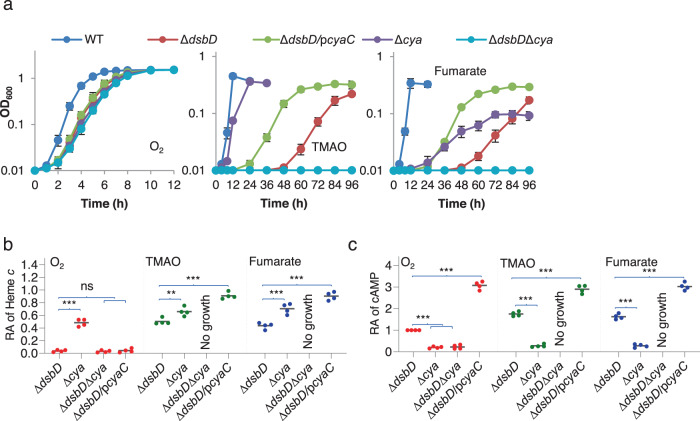
cAMP is involved in the anaerobic growth of the *S. oneidensis dsbD* mutant. **a** Growth of indicated strains on EAs specified. The expression of *cyaC* (p*cyaC*) was driven by IPTG-inducible promoter P*tac*. Shown was the data in the presence of 0.5 mM IPTG for all panels. The experiments were performed four times, and the data were presented as the average ± SD (error bar). **b** Heme *c* levels in strains grown to the early stationary phase. The relative abundance (RA) of heme *c* was obtained as described in Fig. 2b. **c** cAMP levels. After cAMP quantification, the average data of aerobic-growing Δ*dsbD* cells were set to 1 and relative abundance (RA) of cAMP in other samples was calculated out by normalizing it to that of aerobic-growing Δ*dsbD* cells. In **b** and **c**, asterisks indicate statistically significant difference of the values compared (*n* = 4; ns not significant, *p* > 0.05; *P < 0.05; **P < 0.01; ***P < 0.001).

Because the overall cyt *c* content is well correlated with intracellular levels of cAMP, which is present at higher concentrations in cells grown under anaerobic conditions than in those respiring oxygen, we hypothesized that cAMP-Crp may be involved in the observed anaerobic growth of ∆*dsbD*. To test this, the *cyaC* gene, encoding the predominant adenylate cyclase in *S. oneidensis*^26,28^, was expressed at varying levels driven by an IPTG-inducible promoter in ∆*dsbD*. In the presence of 0.5 mM IPTG, growth of the mutant became visible in 24 h, and the cyt *c* content of the cells from 96 h cultures was ~68% relative to that of the wild-type (Fig. 3a, b). Nonetheless, cells grown under aerobic conditions with 1 mM IPTG were still completely defective in CCM (Fig. 3b and Supplementary Fig. 3a). Quantification of cAMP confirmed increased cAMP levels when CyaC was overproduced (Fig. 3c). Consistently, additional deletion of the *cpdA* gene, which encodes the only enzyme that degrades cAMP in *S. oneidensis*^26^, also resulted in elevated cAMP concentrations, leading to improved growth of ∆*dsbD* (Supplementary Fig. 3b). The proposal that elevated cAMP levels play a critical role in rescuing CCM of the ∆*dsbD* strain was further validated by the addition of exogenous cAMP. With respect to growth and the cyt *c* content, the effect of 2 mM cAMP on the *dsbD* mutant was almost the same as that observed from *cyaC* expression with 0.5 mM IPTG (Supplementary Fig. 3b, c). Noticeably, CCM in cells grown under aerobic conditions could not be restored by either CpdA depletion or adding cAMP up to 2 mM, similar to CyaC overproduction (Supplementary Fig. 3a).

To provide additional evidence that cAMP-Crp is linked to the growth of ∆*dsbD* under anaerobic conditions, we removed the *cya* genes (*cyaC* and *cyaA*, which encodes a secondary adenylate cyclase) from the ∆*dsbD* strain^28^. In line with the findings in previous reports^27,28^, strains that could not generate cAMP possess a substantially reduced cyt *c* content and therefore carry varying defects in respiration of oxygen and non-oxygen EAs (Fig. 3a, b). As shown in Fig. 3a, the ∆*dsbD*∆*cya* strain displayed impaired aerobic growth similar to the *cya* mutant and had no detectable cyts *c*. Moreover, ∆*dsbD*∆*cya* was unable to grow on fumarate and TMAO completely, contrasting the *cya* mutant (Fig. 3a). Importantly, the additional removal of Crp from ∆*dsbD* resulted in similar defects in all these aspects (Supplementary Fig. 3a). Based on these data, we conclude that cAMP-Crp plays an essential role in the anaerobic growth of the ∆*dsbD* strain by promoting cyt *c* biosynthesis.

### Screening for genes promoting anaerobic growth of the *dsbD* mutant

Although cAMP-Crp impacts significantly on CCM in the ∆*dsbD* strain, clues for further exploration are scarce because the regulatory system directly or indirectly mediates the expression of hundreds of genes^21^. To identify genes involved in DsbD-independent CCM, a random mutation library was constructed from the ∆*dsbD* strain using mariner-based transposon vector pHGT01, in which a robust promoter is embedded in the transposable sequence^31^. A library of ∼15,000 colonies with transposon insertion formed on LB plates under aerobic conditions were screened for fast-growing strains on TMAO in 96-well plates. In total, 19 strains that exhibited significantly improved growth (e.g., visible growth in 24 h) were obtained.

**Fig. 4:**
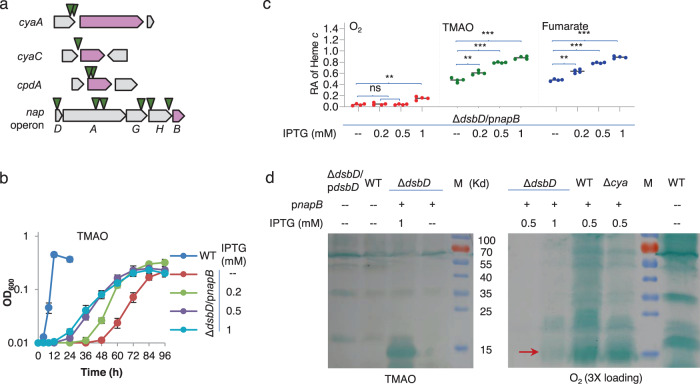
Identification of NapB as the protein facilitating cyt *c* biosynthesis of the *dsbD* mutant. **a** Screening for better growth mutants from ∆*dsbD* by transposon mutagenesis. The transposable element contains robust promoters, aiming at overproducing genes after the insertion. By genetic mapping, 11 random mutants had insertions shown here, which was drawn in scale. Arrows represent the approximate locations of transposon insertions. **b** Impacts of NapB at varying levels on growth on EAs specified. Expression of *napB* (p*napB*) was driven by IPTG-inducible promoter P*tac*. The experiments were performed four times, and the data were presented as the average ± SD (error bar). **c** Heme *c* levels in strains grown to the early stationary phase. The relative abundance (RA) of heme *c* was obtained as described in Fig. 2b. Asterisks indicate statistically significant difference of the values compared (*n* = 4; ns not significant, *p* > 0.05; *P < 0.05; **P < 0.01; ***P < 0.001). **d** Heme-staining. Samples prepared the same as for quantification of heme *c* were processed, protein contents were quantified, and the equal amounts of proteins were separated in SDS-PAGE, and then subjected to heme-staining. ∆*dsbD*/p*dsbD*, complemented ∆*dsbD*, M molecular weight marker. NapB was indicated by arrows. Aerobic growth samples were overloaded (3X) for better visualization.

Insertion sites in 11 random mutation strains were successfully identified. Two and one were found to have transposon insertions in the regions upstream of adenylate cyclase genes *cyaA* and *cyaC* respectively (Fig. 4a). As no insertions in the coding sequences of these two genes were caught, this observation suggests that both *cyaC* and *cyaA* genes are likely overexpressed because of the transposon-borne promoter. Additionally, two isolates had transposons that mapped within the coding sequence of the *cpdA* gene (Fig. 4a), apparently destroying the function. These data are in perfect agreement with the finding that cAMP at increased concentrations could partially suppress the CCM defect of the ∆*dsbD* strain.

Surprisingly, six isolates had transposons that mapped in the region of the *napDAGHB* operon, which encodes periplasmic nitrate reductase and accessory proteins (Fig. 4a). In *S. oneidensis*, only NapA (the catalytic subunit of the reductase) is essential for nitrate respiration because cyt *c* quinol dehydrogenase CymA, in place of otherwise essential NapC, transports electrons from the quinol pool to NapA, via NapB if it is present^32^. Given that two insertions are within the *napA* gene, nitrate reduction is apparently irrelevant to CCM of the ∆*dsbD* strain. Although these insertions within the locus appear random, the transposon was never identified in the *napB* coding sequence, implying a possibility that expression but not the function of the *napB* gene is affected by the insertions. To test this, we manipulated *napB* expression in the ∆*dsbD* strain and assessed its effect on the CCM defect. In the presence of 0.2 mM IPTG, *napB* expression in trans significantly elevated cyt *c* levels and improved growth of the ∆*dsbD* strain on TMAO or fumarate (Fig. 4b, Supplementary Figure 4). Cyt *c* production of the ∆*dsbD* strain expressing the *napB* gene was further improved with higher concentrations of IPTG, up to 1 mM (Fig. 4c). In terms of growth, NapB overproduction mainly shortened the lag time (Fig. 4b and Supplementary Fig. 4). Induced production of NapB in the ∆*dsbD* strain was validated by using heme-staining; clearly, NapB levels increased with IPTG concentrations (Fig. 4d). Despite substantially induced production NapB in the presence of 1 mM IPTG, the overall cyt *c* content of the ∆*dsbD* strain was still significantly lower than that of the wild-type (Fig. 4c). Notably, the ∆*dsbD* strain with NapB overproduction by 1 mM IPTG was able to produce a residual quantity of cyts *c* in oxygen-respiring cells (Fig. 4c, d). Altogether, these data conclude that NapB, when overproduced, is able to suppress the CCM defect resulting from the loss of DsbD under anaerobic conditions in *S. oneidensis*.

### NapB underlies the effect of cAMP on CCM of the ∆*dsbD* strain during anaerobiosis

**Fig. 5:**
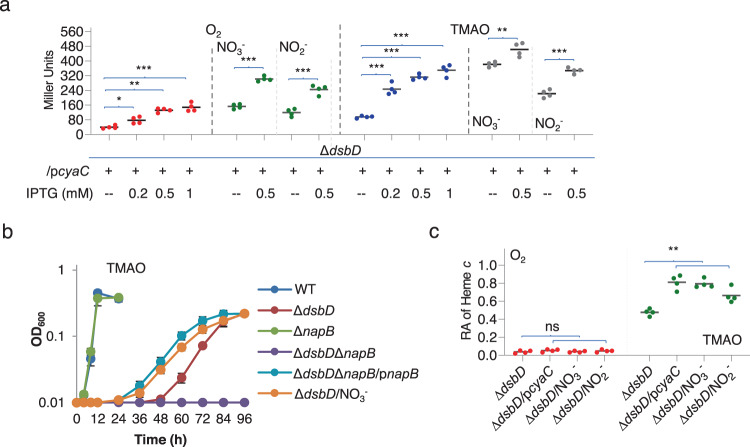
NapB underlies the effect of cAMP on cyt *c* biosynthesis of the *dsbD* mutant. **a** Expression of *napB*. Samples were prepared with cells of indicated strains grown to the exponential phase. The promoter activity of *nap* was assayed by an integrative *lacZ* reporter. **b** Growth of indicated strains on EAs specified. The expression of *cyaC* (p*cyaC*) was driven by IPTG-inducible promoter P*tac*. Shown was the data in the presence of 0.2 mM IPTG for all panels. The experiments were performed four times, and the data were presented as the average ± SD (error bar). **c** Heme *c* levels in strains grown to the early stationary phase. The relative abundance (RA) of heme *c* was obtained as described in Fig. 2b. In a and c, asterisks indicate statistically significant difference of the values compared (*n* = 4; ns not significant, *p* > 0.05; *P < 0.05; **P < 0.01; ***P < 0.001).

As demonstrated above, growth and CCM of the ∆*dsbD* strain can be promoted by elevated concentrations of cAMP and overproduced NapB. The *napB* gene is transcribed only by the promoter for the *nap* operon, which is a member of the Crp regulon in *S. oneidensis*^29,33^. To assess the impacts of cAMP on *napB* expression, we monitored the *nap* promoter with an integrative *lacZ* reporter in the presence of cAMP at varying concentrations, which were manipulated by IPTG-controlled CyaC production (Fig. 5a). During aerobiosis, the *nap* operon was merely expressed but became active in cells grown on TMAO. When cAMP was forcibly produced in cells grown on O_2_ or TMAO, the activity of the *nap* promoter increased with cAMP levels, with more than five times induction in the presence of IPTG at 0.5 mM or higher (Fig. 5a). Based on these observations, we proposed that the rescuing effect of elevated cAMP levels may be a consequence of the induced expression of the *napB* gene.

The transcription of the *S. oneidensis nap* operon is also under the control of the two-component system NarQP, which is responsive to both nitrate and nitrite^29^. This regulation implies a possibility that nitrate/nitrite boosts NapB production and therefore promotes CCM of the *dsbD* mutant. Indeed, in the presence of 1 mM nitrate or 0.5 mM nitrite (below the growth inhibitory concentrations^34^) the *nap* promoter exhibited a significant increase in activity in cells grown not only on TMAO but also on oxygen (Fig. 5a). Although impacts of nitrate/nitrite addition on growth and CCM were observed only in cells grown under anaerobic conditions (Fig. 5a, c and Supplementary Fig. 5a, b), it is clear that nitrate/nitrite, by inducing NapB, can alleviate the CCM defect of the *dsbD* mutant during anaerobiosis.

To provide direct evidence concerning the involvement of NapB, we removed the *napB* gene from the ∆*dsbD* strain and assessed the impacts of elevated cAMP levels on the growth of the resultant double mutant. In the absence of NapB, the ∆*dsbD* strain was unable to grow on TMAO with CyaC overproduction by up to 1 mM IPTG (Fig. 5b). When a copy of *napB* was expressed in trans, the ∆*dsbD*∆*napB* strain recovered the ability to grow on TMAO (Fig. 5b). NapB has been shown to be one of several soluble cyts *c* compromising extracellular electron transfer by promiscuously dissipating electrons obtained from the quinol pool to other electron-accepting agents in the periplasm^35–37^. We then tested whether the CCM-rescuing effect of NapB is a common feature shared by all of these soluble cyts *c* with two representatives, FccA and TsdB. However, when overexpressed, neither of the two proteins was able to rescue growth of the ∆*dsbD*∆*napB* strain on TMAO (Supplementary Fig. 5c). These data indicate that NapB owns a distinct activity required for rescuing the CCM of the ∆*dsbD* strain.

### Modulation of CCM by NapB in the *dsbD* mutant depends on CcmG

**Fig. 6:**
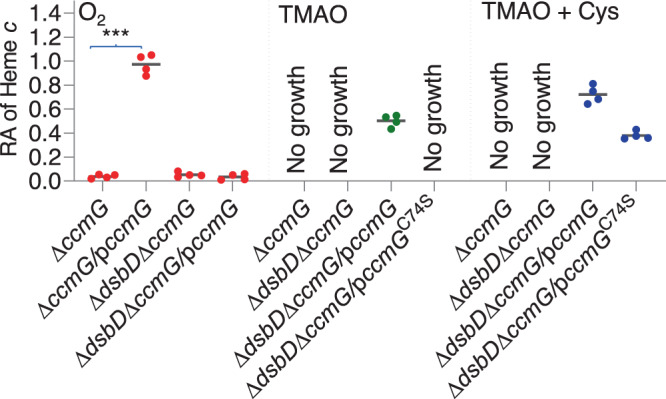
Modulation of cyt *c* biosynthesis by NapB in the *dsbD* mutant depends on CcmG. Heme *c* levels in indicated strains grown to the early stationary phase. The relative abundance (RA) of heme *c* was obtained as described in Fig. 2b. TMAO + Cys, cultures supplemented with 5 mM Cysteine. Asterisks indicate statistically significant difference of the values compared (*n* = 4; *P < 0.05; **P < 0.01; ***P < 0.001).

In bacteria with a Ccm system, DsbD transfers electrons from intracellular donors via CcmG and CcmH to CcmF, resulting in the reduction of oxidized apocyts *c* such that ligation of heme *b* to free thiol of apocyt *c* could occur^1^ (Fig. 1). Given that NapB functions to transport electrons from CymA to terminal reductases^32,33^, we hypothesized that it may deliver electrons to Ccm components when DsbD is absent. To test this, we determined the essentiality of redox-active Cys residues within CcmG for activity as the protein is the immediate electron acceptor for DsbD (Fig. 1). Like DsbD and CcmF, CcmG was essential for CCM in *S. oneidensis*: the *ccmG* mutant was neither able to grow on TMAO nor able to produce any cyt *c* during aerobiosis, and these phenotypes were verified by genetic complementation with wild-type CcmG (Fig. 6). In contrast, CcmG^C74S^, a mutant in which redox-active residue Cys74 is replaced by serine and thus loses electron-transfer ability^38^, did not show any detectable effect on growth on TMAO or CCM during aerobiosis. Thus, NapB requires CcmG for its rescuing effect on CCM of the *dsbD* mutant.

We then removed *dsbD* from the CcmG-deficient background, generating double mutant ∆*dsbD*∆*ccmG*, which, not surprisingly, behaved identically as the ∆*ccmG* strain (Fig. 6). Expression of the wild-type CcmG corrected the phenotype resulting from the CcmG loss, making this double mutant grow on TMAO and produce cyts *c* the same as the ∆*dsbD* strain. On the contrary, the complementation of ∆*dsbD*∆*ccmG* by CcmG^C74S^ failed to restore growth on TMAO (Fig. 6). These data suggest that NapB suppresses the CCM defect of the ∆*dsbD* strain by functioning as an electron donor to CcmG for reduction of oxidized apocyts *c*.

### NapB functions differently from small molecule reducing agents

**Fig. 7:**
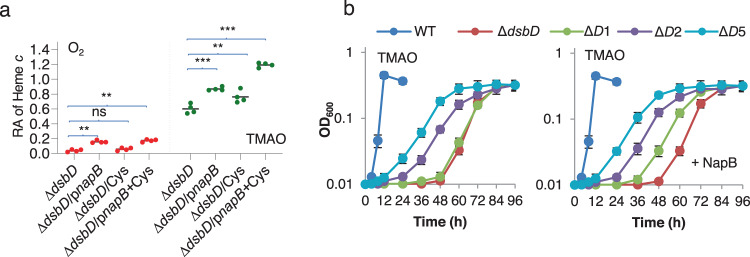
Small reducing agents differ from NapB in modulating cyt *c* biosynthesis in the *dsbD* mutant. **a** Heme *c* levels in indicated strains grown to the early stationary phase. NapB expression was achieved in the presence of 1 mM IPTG. The relative abundance (RA) of heme *c* was obtained as described in Fig. 2b. Asterisks indicate statistically significant difference of the values compared (*n* = 4; ns not significant, *p* > 0.05; *P < 0.05; **P < 0.01; ***P < 0.001). **b** Growth of indicated strains on EAs specified. ∆*D*1, ∆*D*2, and ∆*D*5, represent that ∆*dsbD* grew in the presence of 1, 2, 5 mM cysteine respectively. NapB expression was achieved in the presence of 0.2 mM IPTG. The experiments were performed four times, and the data were presented as the average ± SD (error bar).

A prerequisite for NapB to provide electrons for reduction of oxidized apocyts *c* is the presence of matured NapB proteins, which per se is a cyt *c*. The most likely explanation would be that a residual number of apocyt *c* molecules exist in reduced form and thereby can mature independent of DsbD during anaerobiosis. If this holds true, we hypothesized that small-molecule reductants that increase the quantity of reduced apocyts *c* prior to the Ccm system may also facilitate CCM in the ∆*dsbD* strain (Fig. 1). To test this, we measured growth and cyts *c* levels of ∆*dsbD* supplemented with reductants L-cysteine and reduced GSH at varying concentrations substantially higher than endogenous ones. The normal level of intracellular cysteine in growing *E. coli* is up to 0.2 mM and GSH concentrations could be ten times higher but their concentrations in the periplasm are conceivably lower^39–41^. Cysteine at up to 5 mM (inhibiting growth at higher concentrations) did not elicit a noticeable impact on growth and the cyt *c* content of ∆*dsbD* under aerobic conditions (Fig. 7a and Supplementary Fig. 6). However, in its presence, growth on TMAO and CCM of the ∆*dsbD* strain were significantly improved (Fig. 7a, b). Cysteine addition clearly had more prompt impacts on growth of the ∆*dsbD* strain than NapB overproduction, evidenced by substantially shortened lag times (Fig. 7b). Despite this, the cyt *c* content of the ∆*dsbD* strain with 2 mM cysteine was still at least 40% lower than that in the wild-type. Similar results were obtained from reduced GSH (Supplementary Fig. 7).

To investigate whether cysteine functions the same as NapB, we assessed its effect on the ∆*dsbD*∆*ccmG* strains expressing CcmG variants. Expectedly, in the presence of 5 mM cysteine, the ∆*dsbD*∆*ccmG* strain could not grow on TMAO at all (Fig. 6). However, in contrast to NapB, cysteine was able to improve growth and CCM when CcmG^C74S^ was present, indicating that its effect is independent of electron transfer via CcmG. This difference provides further support that NapB reduces oxidized apocyts *c* by passing electrons to CcmG (Fig. 6).

As small reducing molecules and NapB function at different stages of CCM, we speculate that their effects on the ∆*dsbD* strain would be additive. Indeed, in the presence of 2 mM cysteine, NapB produced with 0.2 mM IPTG fully restored biosynthesis of cyt *c* of the ∆*dsbD* strain to the wild-type capacity (Fig. 7a and Supplementary Fig. 1). In parallel, growth rates were significantly accelerated by NapB production (Fig. 7b). It is worth mentioning that 5 mM cysteine modestly slowed aerobic growth of both wild-type and the ∆*dsbD* strain but did not show any effect on CCM, even when NapB was overproduced (Figs. 6, 7b and Supplementary Fig. 1). These data collectively indicate that NapB functions differently from small molecule reducing agents although the latter can also partially improve the CCM resulting from the DsbD loss when non-oxygen EAs are respired.

### Only β-type NapB can rescue the CCM defect of ∆*dsbD*

**Fig. 8:**
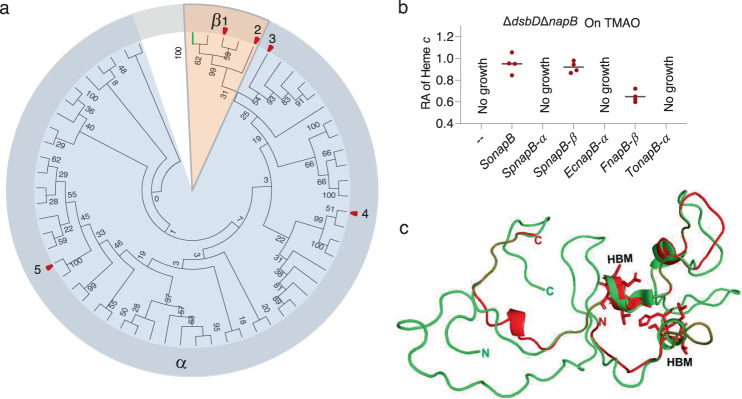
Comparison of α- and β-type NapB proteins. **a** Phylogenetic analysis of NapB proteins. The evolutionary history was represented by the bootstrap consensus tree inferred from 1000 replicates using the Neighbor-Joining method. Bold green line represents the position *of S. oneidensis* NapB. Red arrows represent NapBs that were examined for their effect on cyt *c* biosynthesis. The tree with bacterial names was given in Supplementary Fig. 8. (1) *S. piezotolerans* (β); (2) *Ferrimonas* sp. S7 (β); 3, *Thiosymbion oneisti*; 4, *E. coli*; 5, *S. piezotolerans* (α). **b** Heme *c* levels in Δ*dsbD*Δ*napB* expressing one of *napB*s as indicated grown on TMAO to the early stationary phase. The relative abundance (RA) of heme *c* was obtained as described in Fig. 2b. **c** Structural comparison of *R. sphaeroides* and predicted *S. oneidensis* NapB proteins. Shown is a superimposition of *Sp*NapB (PDB id 1ogy, red) and *So*NapB (green).

In most *Shewanella* species, two NapB paralogs (α-type and β-type) are present which are encoded by genes within *napEDABC* and *napDAGHB nap* operons respectively, but *S. oneidensis* contains β-type NapB only^42^. *Shewanella* β-type NapB proteins are highly similar, sharing ∼60% sequence identity (amino acid), whereas it is ~45% for the α-type. Notably, the sequence conserveness of the second half (∼65 to the C-terminal end) of α-NapB proteins, which contains two HBM sites and additional His residues essential for heme orientation, is substantially higher than that of the first half (Supplementary Fig. 8a, b). The difference in amino acid sequence between the two different NapB isoforms is expectedly more profound (identity <30%) (Supplementary Fig. 8c). In line with this, in homology alignments, *Shewanella* NapB proteins and their corresponding orthologs in other bacteria form two distinct clades (Fig. 8a and Supplementary Fig. 9). Moreover, we found that α-type NapB proteins are notably larger (158 a.a. on average) than their β-type counterparts (142 a.a. on average).

These differences between NapB isoforms prompted us to speculate whether α-type NapB proteins could also be able to serve as an electron donor for CcmG, resulting in DsbD-independent CCM. To test this, we examined the effects of NapB proteins from *S. putreficiens* (both α- and β-type), *E. coli* (α-type), *Ferrimonas* sp. S7 (β-type), and *Thiosymbion oneisti* (α-type), which belong to different subclades, in the ∆*dsbD*∆*napB* strain under TMAO-growing conditions (Fig. 8a and Supplementary Fig. 9). As expectedly, β-type NapB of *S. putreficiens* generated an impact indistinguishable from that of the *S. oneidensis* NapB (Fig. 8b). Similar results were obtained from NapB of *Ferrimonas* sp. S7. In contrast, the production of any α-type NapB under test did not show any detectable effect (Fig. 8b). As *T. oneisti* NapB represents an α-type NapB being the closest to the β-type in phylogeny, these data imply that only β-type NapB proteins are capable of rescuing the CCM defect resulting from the DsbD loss.

We then utilized computational modeling to assess structural differences between these two types of NapB proteins. To date, structures of three bacterial NapB proteins are available (PDB id 1ogy, *Rhodobacter sphaeroides*; 3o5a, *Cupriavidus necator*; and 1jni, *Haemophilus influenzae*), but all belong to the α-type^43–45^. All of these NapBs are structurally similar, especially in the heme-attachment region (the second half) composed of three short α-helices that are connected by large loops^46^ (Fig. 8c and Supplementary Fig. 9). The structure of *S. oneidensis* NapB was then predicted and consistent with the sequence conserveness, *So*NapB (β-type) differs from others (α-type) in N-terminal segments (the first half) (Fig. 8c and Supplementary Figs. 8, 10), suggesting a possibility that the N-terminal half may play a particularly important role in defining CcmG-reducing activity of β-type NapB proteins. Furthermore, we hypothesized that all of the differences between two types of NapB proteins described above may affect midpoint redox potentials, which may be critical for NapB to reduce CcmG. To this end, we expressed recombinant *So*NapB^strep^ from *E. coli* and determined its midpoint redox potentials to be −30 and −190 mV approximately (Supplementary Fig. 11). These values, similar to midpoint redox potentials of α-type NapB proteins known to date, including −25 and −170 mV for *Haemophilus influenzae* NapB^47^, −100 and −200 mV for *R. sphaeroides* NapB^44^, and −10 and −180 mV for *E. coli* NapB^48^, are in a range that is commonly found for bis-His-ligated hemes that have low redox potentials as a result of the electron-donating properties of the imidazole ring nitrogens, which serve to stabilize the ferric state. Thus, given that the midpoint redox potentials of two types of NapB proteins are in similar ranges, there must be other differences between them that determine the CcmG-reducing activity of β-type NapB proteins.

## Discussion

*Shewanella* are renowned for their respiratory versatility, which lays the foundation for their enormous potential in bioenergy and bioremediation^17^. The unparallel capability for utilization of diverse EAs is based on a vast number of cyts *c* although a strategy is evolved to respire oxygen independent of cyts *c*^19^. The fact that cyts *c* have to go through a particular maturation process brings out an eminent threat: its inactivation would wipe out all cyts *c* encoded in the *S. oneidensis* genome. Seemingly, multiple strategies have evolved to prevent this tragedy from the occurrence. Genes for the Ccm components of *Shewanella* are in proximity but arranged into multiple operons, each of which carries out a distinct function, such as heme transport, apocyt *c*-heme ligation, and yet fully defined one (CcmI)^1,22^. In contrast to the single-operon organization for Ccm genes in most other γ-proteobacteria, this arrangement conceivably renders cells more resilient to mutations. To prevent apocyts *c* in the reduced form to be unnecessarily degraded, multiple DsbAs and DsbBs are in place to promptly oxidize apocyts *c* once they are in the periplasm, as revealed in *S. oneidensis*^7^ (Fig. 1). This oxidation process is reinforced by oxygen and/or yet-identified oxidizing agents during anaerobiosis because the loss of all functional DsbAs does not completely abolish CCM. In the meantime, previous studies had shown that the intracellular redox systems (thioredoxin (Trx) and glutaredoxin (Grx) systems) and auxiliary redox proteins are also intertwined, potentially offering multiple routes for delivering electrons to DsbD^49,50^.

The finding that the DsbD depletion completely annuls CCM in *S. oneidensis* cells respiring oxygen but not non-oxygen EAs indicates that a share of cyts *c* can be matured in the absence of DsbD, at least when growth takes place under anaerobic conditions. Given that the DsbD family consists of proteins belonging to multiple classes, including the DsbD-like, CcdA-like, and ScsB-like^51,52^, we anticipated that the genome may encode additional homologs of these proteins. However, this was not the case. Neither additional DsbD homologs nor homologs of CcdA-like and ScsB-like proteins are present in *S. oneidensis*. Instead, NapB was identified to partially rescue CCM in the *dsbD* mutant. This behavior of NapB, to our knowledge, is unprecedented because the protein per se is a cyt *c*. Undoubtedly, there is an evident prerequisite for this strategy to function: DsbD-independent maturation of NapB at the first place, albeit most likely only in a tiny amount.

It is certain that cells respiring oxygen and non-oxygen EAs bear a substantial difference in robustness of apocyt *c* oxidation in the periplasm^6,53^. In aerobic environments, oxygen is a predominant force to drive oxidation of apocyts *c* in *S. oneidensis*^7^. The notion is clearly echoed by the finding presented here that oxygen is so powerful that it effectively prevents NapB maturation in the *dsbD* mutant even when exogenous reducing molecules are present. In the absence of oxygen, however, a residual quantity of reduced apocyts *c*, including NapB, manage to go through the maturation process without needing reducing equivalents from DsbD. We propose that reduced apo-NapB is particularly resilient to degradation, enhancing odds to be matured.

The data presented here establish the order of thioreduction reactions between NapB and Ccm components, which is essential for elucidating the mechanism for NapB’s impacts on the CCM defect resulting from the DsbD loss (Fig. 1). DsbD conveys reducing equivalents to CcmG, the first component of the Ccm-specific thioreductive pathway that reduces the disulfide bond in oxidized apocyts *c* to allow covalent heme ligation^54^. Although CcmG may play an additional role in chaperoning apocyts *c*, its primary responsibility is to efficiently mediate electron transfer from DsbD to CcmH, which forms the heme ligation complex with CcmF, and CcmI if available^38,55,56^. The observation that NapB has no effect in the ∆*dsbD*∆*ccmG*/CcmG^C75S^ strains manifests that the action of NapB is to pass electrons to CcmG. In contrast, reducing agents function independently of electron transfer activity of CcmG, indicating that they possibly counteract apocyt *c* oxidation and/or reduce CcmH (Fig. 1). Despite this, reducing agents may also be capable of reducing NapB in the absence of quinol dehydrogenases through which NapB obtains electrons from the quinol pool. Either way, the amount of matured cyts *c* is substantially augmented.

Although NapB is the determining factor for rescuing CCM in the *dsbD* mutant, it is cAMP that differentiates the physiological impact of NapB on CCM in cells grown with or without oxygen. As a master regulator mediating respiration, upon binding to cAMP-Crp directly activates transcription of many cyt *c* genes, including the *nap* operon^19,21,25,29^. To date, enzymatic sources for cAMP biosynthesis in *S. oneidensis* are known^28^, but how the adenylate cyclases modulate intracellular cAMP levels in response to environmental cues remains elusive. Despite this, it is clear that there are significantly more cAMP molecules in cells respiring non-oxygen EAs than those respiring oxygen^19,21,33^. As a result, transcription of the *napB* gene (within the *nap* operon) is allowed in cells grown anaerobically. Given that the *nap* promoter appears inactive in oxygen-respirating cells, we speculate that in aerobic habitats cAMP concentrations may not exceed the threshold that Crp activation requires. Transcription of the *nap* operon is also mediated by NarQP TCS that is responsive to both nitrate and nitrite^29^. Through this dual-level control mechanism, cAMP determines whether environments are suitable for *nap* expression while NarQP promotes expression if the inducers are present^29^.

Unexpected involvement of NapB in CCM convinces us that evolution has honed *Shewanella* to proliferate in redox-stratified habitats. Proteomics studies have demonstrated that a large majority of cyts *c* are present in cells grown on oxygen or non-oxygen EAs^57,58^. We speculate that this is a result of cAMP-mediating respiratory regulation through which the expression of cyts *c* fluctuates with the levels of intracellular cAMP^19,33^. As cyts *c* are essential for anaerobic growth but only confer cells a limited advantage to oxygen respiration, the restoration of CCM by NapB is designed to kick in in anaerobic habitats only. Importantly, this mechanism likely occurs naturally because of the inducible production of NapB by the most common nitrogen species, nitrate and nitrite, which are ubiquitous in global waterbodies, let alone redox-stratified niches.

NapB proteins that can rescue CCM in the *dsbD* mutant belong to β-type exclusively, which are present in a small portion of bacteria hosting periplasmic nitrate reductases. The two NapB isoforms are rather conserved in the second half region (making up ∼60%) covering two heme-attachment sites, but vary substantially in the first half region in both sequence and structure. NapB of α-type exists in the form of NapAB heterodimer whereas its β-type counterpart is not closely associated with NapA^59^. This likely explains why NapBs of α-type but not β-type are essential for nitrate reduction^32,60^. Moreover, β-type NapBs could function as a promiscuous electron donor, delivering electrons to multiple yet unidentified electron-accepting agents^24,35–37^. Although this promiscuity has been observed from several cyts *c* in *S. oneidensis*, only NapB is able to rescue CCM in the *dsbD* mutant grown under anaerobic conditions^35–37^. Based on this understanding, it is not surprising that CcmG could be reduced by NapB.

It should be noted that β-type NapB proteins do not differ from their α-type counterparts in midpoint redox potentials (above −190 mV) significantly and these midpoint redox potentials seem to hamper reduction of CcmG whose redox potential is between −220 and −175 mV^61–64^. However, the thermodynamic feasibility of oxidation-reduction is not dependent on the redox potential only. For example, CymA, containing four hemes with redox potentials of −110, −190, −240 and −265 mV respectively, transfers electrons from menaquinol to various terminal electron acceptors and could be reduced with menadiol (E_m_ = −80 mV) in the presence of NADH (E_m_ = − 320 mV) and an NADH–menadione oxidoreductase. It has been proposed that thermodynamically unfavorable reductions may be overcome in the cellular context by its integration with an electrical circuitry that can regulate this electron flux at the levels of both electron supply and demand^23^. In the disulfide bond redox system, DsbB is responsible for reoxidizing DsbA although the redox potentials of the two cysteine pairs of DsbB are −207 and −224 mV respectively and DsbA has a redox potential of −119 mV. This thermodynamically unfavorable reaction becomes possible due to certain changes in the structure of DsbB^65^. Overall, whether a thermodynamical reaction can occur depends not on the redox potential of protein only.

In addition to the significant differences between α- and β-type NapBs in terms of biochemical properties, whether *napC* is present or not in the *nap* operon may add another dimension to the distinct of two NapB isoforms. Among bacterial *nap* gene clusters, *nap-*α (*napEDABC*) and *nap-*β (*napDAGHB*) operons are most common and the sharply different gene arrangements within them imply that they may evolve independently from each other^42^. Genes for β-type NapB never co-exist with *napC*, encoding a tetraheme, membrane-bound cyt *c* which is the quinol oxidase specifically mediating electron transport from the quinol pool to the α-type NapAB complex^42^. Thus, it is reasonable to infer that the promiscuity of β-type NapBs for electron acceptors may be a result of the lack of NapC. To take this a step further, β-NapBs may also be promiscuous for its electron donors. Indeed, *S. oneidensis* NapB can also receive electrons from quinol dehydrogenase the cyt *bc*_1_ complex (PetABC), in addition to CymA^32,66^. Given that CymA and PetABC per se are cyt *c* proteins, which may not be produced in the absence of DsbD, we propose that non-cyt *c* electron donors, small-molecule reducing agents, cysteine and GSH in particular, probably fulfill this role.

## Methods

### Bacterial strains, plasmids, and culture conditions

All bacterial strains and plasmids used in this study were listed in Table 1. Information for primers used in this study was given in Supplementary Table 1. For genetic manipulation, *E. coli* and *S. oneidensis* were grown in Lysogeny broth (LB, Difco, Detroit, MI) under aerobic conditions at 37 ^o^C and 30 ^o^C, respectively. When appropriate, the growth medium was supplemented with chemicals at the following final concentrations: 2, 6-diaminopimelic acid (DAP), 0.3 mM; ampicillin, 50 μg/ml; kanamycin, 50 μg/ml; gentamycin, 15 μg/ml; and streptomycin, 100 μg/ml.

**Table 1:** Strains and plasmids used in this study.

Strain or plasmid	Description	Reference or source
Strains *E. coli*		
BL21(DE3)	Host for expression	Novagen
WM3064	Donor strain for conjugation, ∆*dapA*	W. Metcalf, UIUC
*S. oneidensis*		
MR-1	Wild-type	ATCC 700550
HG0266	∆*ccmF* derived from MR-1	(3)
HG0696	Δ*dsbD* derived from MR-1	(30)
HGCYA	∆*cya* (Δ*cyaA*Δ*cyaB*Δ*cyaC*) derived from MR-1	(26)
HG0696-CYA	∆*dsbD*∆*cya* derived from MR-1	This study
HG0845	∆*napB* derived from MR-1	(32)
HG0696-0845	∆*dsbD*∆*napB* derived from MR-1	This study
HG0267	∆*ccmG* derived from MR-1	This study
HG0696-0267	∆*dsbD*∆*ccmG* derived from MR-1	This study
HG0265	∆*ccmI* derived from MR-1	(22)
HG0696-3901	∆*dsbD*∆*cpdA* derived from MR-1	This study
HG0624	∆*crp* derived from MR-1	(21)
HG3901	∆*cpdA* derived from MR-1	(26)
Plasmids		
pHGM01	Ap^r^, Gm^r^, Cm^r^, *att*-based suicide vector	(3)
pHGEI01	Integrative *lacZ* reporter vector	(67)
pHGNE-P*tac*	Km^r^, IPTG-inducible P_*tac*_ expression vector	(69)
pHGT01	Promoter-embedded transposon vector	(31)
pRSFDuet-1	expression vector co-expressing two target ORFs, Km^r^	Novagen
pHGNE-P*tac*-*gene*	IPTG-controlled expression of genes, including *ccmF*, *napB*, *dsbD*, *cyaC*, *ccmG*, *ccmG*^C74S^, *α*-*napB* from *S. putreficiens*, *β*-*napB* from *S. putreficiens*, *α*-*napB* from *E. coli*, *β*-*napB* from *Ferrimonas sp. S7*, *α*-*napB* from *T. oneisti*, and all genes for genetic complementation	(3,26,32,34) and This study
pHGEI01-P*nap-lacZ*	Vector for measuring P_*nap*_ activity	This study
pRSFDuet-1-*Ec*Ccm-*So*NapB^strep^	pRSFDuet-1 expressing the *E. coli* Ccm system and *So*NapB^strep^	This study

Growth of *S. oneidensis* strains under aerobic or anaerobic conditions was determined by recording the optical density of cultures at 600 nm (OD_600_). MS defined medium [KCl, 1.34 mM; NaH_2_PO_4_, 5 mM; Na_2_SO_4_, 0.7 mM; NaCl, 52 mM; piperazine-*N*,*N* = -bis(2-ethanesulfonic acid) (PIPES), 3 mM; NH_4_Cl, 28 mM; sodiumlactate, 30 mM; MgSO_4_, 1 mM; CaCl_2_, 0.27 mM; and FeCl_2_, 3.6 μM, pH 7.0] containing 30 mM lactate as electron donor was used as previously described^67^. For aerobic growth, mid-log cultures were inoculated into fresh medium to an OD_600_ of ∼0.05 and shaken at 200 rpm at 30 ^o^C. For anaerobic growth, cultures were purged with nitrogen and inoculated into fresh media prepared anaerobically to an OD_600_ of ∼0.05 containing fumarate (20 mM) or TMAO (20 mM).

### In-frame mutant construction and complementation

In-frame deletion strains for *S. oneidensis* were constructed using the *att*-based fusion PCR method as described previously^3^. In brief, two fragments flanking gene of interest were amplified by PCR and joined together by the second round of PCR. The fusion fragments were introduced into plasmid pHGM01 by using Gateway BP clonase II enzyme mix (Invitrogen) according to the manufacturer’s instruction, and the resulting mutagenesis vectors were transformed into in DAP-auxotroph *E. coli* WM3064. After verification, the vectors were transferred from WM3064 into *S. oneidensis* via conjugation. Integration of the mutagenesis constructs into the chromosome was selected by resistance to gentamycin and confirmed by PCR. Verified transconjugants were grown in LB broth in the absence of NaCl and plated on LB supplemented with 10% sucrose. Gentamycin-sensitive and sucrose-resistant colonies were screened by PCR for deletion of the target gene. Mutants were verified by sequencing the site for intended mutation.

The majority of mutants used in this study are previously constructed and verified by genetic complementation (Table 1). For newly constructed mutants, plasmid pHGEN-P*tac* that carries IPTG-inducible promoter P_*tac*_ was used in genetic complementation^68^. The coding sequence of the target genes was amplified and inserted into after P_*tac*_. After sequencing verification, resulting vectors were transferred into the relevant strains via conjugation for complementation. In all experiments with strains expressing a gene within a plasmid, all other strains for comparison carried a copy of the empty plasmid.

### Transposon mutagenesis

A random mutation library for *S. oneidensis* was constructed with pHGT01, which is a transposon vector with a strong promoter embedded in the transposable region^31^. A library of ∼15,000 colonies with transposon insertion formed on LB plates under aerobic conditions were transferred MS medium with TMAO in 96-well plates prepared anaerobically. Suppressors for the *dsbD* deletion were selected by visible growth in 24 h in the presence of gentamycin. Suppressors were subjected to the mapping of the transposon insertion sites by using the arbitrary PCR^69^.

### Chemical assays

Unless otherwise noted, cultures of the early stationary phase were used for chemical assays. Cyt *c* abundance of strains was first estimated by the color intensity of cell-pellets. When necessary, heme *c* levels were quantified with the QuantiChrom heme assay kit (BioAssay Systems) according to the manufacturer’s instructions. Cells were collected by centrifugation and suspended in PBS buffer. The OD_600_ of each cell suspension was adjusted to 1 and total bound heme in 50 μl of cell suspension aliquot for each strain was determined.

To determine cAMP (cyclic adenosine 3′,5′-monophosphate) concentrations, cultures were subjected to filtering through a 0.22 µm nylon membrane for the separation of cells and cell-free filtrate. The filtrate was immediately for cAMP assay, which was performed by using a cAMP direct immunoassay kit (BioVision) according to the manufacturer’s instructions. The external cAMP levels were used to estimate the cAMP excretion rate by multiplying the specific growth rate and normalizing to OD_600_ values as described elsewhere^33^. The relative cAMP excretion rate for each mutant strain was given by comparing to that of the wild-type, representing the relative internal cAMP level. Standard curves were made with commercial agents each time.

### Promoter activity assay

The activity of promoters of interest was assessed using a single-copy integrative *lacZ* reporter system as described previously^66^. A fragment covering the sequence upstream of each operon tested from −300 to +1 was then amplified and cloned into the reporter vector pHGEI01, verified by sequencing, and the correct plasmid was then transferred into relevant *S. oneidensis* strains by conjugation. Once transferred into *S. oneidensis* strains, pHGEI01 containing the promoter of interest integrates into the chromosome and the antibiotic marker is then removed by an established approach^25^. Cells in the log phase (OD_600_ = 0.6) were harvested by centrifugation, washed with PBS (phosphate-buffered saline), and treated with lysis buffer. The resulting soluble protein was collected after centrifugation and used for enzyme assays employing the High Sensitivity b-Galactosidase Assay Kit (Stratagene) according to the manufacturer’s instructions. β-Galactosidase activity was determined by monitoring color development at 575 nm every minute for 30 min by using a Synergy 2 Multi-Detection Microplate Reader. The protein concentration of the cell lysates was determined using a Bradford assay with BSA as a standard (Bio-Rad).

### Heme-staining assays

Cells of the early stationary phase were harvested by centrifugation, washed with phosphate-buffered saline (PBS), resuspended in the same buffer, and sonicated. Protein concentrations of the cell lysates were determined by the bicinchoninic acid assay (Pierce Chemical). For heme-staining, the cell lysates were separated on SDS-PAGE using 12% polyacrylamide gels and stained with 3,3′,5,5′-tetramethylbenzidine (TMBZ) as described elsewhere^22^.

### Expression, purification, and spectropoentiometric characterization of NapB

*S. oneidensis* NapB proteins was purified as Strep-tagged soluble proteins from *E. coli* BL21(DE3). DNA fragments for *Ecccm* (encoding the entire Ccm system) and *SonapB* were cloned into pRSFDuet-1 expression vector to give Strep-tagged NapB, which was expressed with 50 μg/mL 5-aminolevulinic acid, whose synthase is the rate-limiting enzyme of heme synthesis. After induction by 1 mM IPTG for 16 h at 16 °C, 5 L cells were harvested and resuspended in buffer containing 20 mM Tris-HCl and 200 mM NaCl. The periplasmic protein fraction, obtained by incubating cells with polymyxin B (1 mg/ml) at 37 °C for 1 h and centrifuging the resultant at 15,000×*g* at 4 °C for 30 min, was loaded onto a 5 ml StrepTrap HP column (GE Healthcare) and *So*NapB^Strep^ was eluted by 2.5 mM desthiobiotin. Proteins were analyzed by 12% SDS-PAGE, followed by staining with Coomassie brilliant blue R250. Identification of purified proteins was confirmed with LC-MS/MS analysis as described before^70^.

UV-visible absorption spectra of purified *So*NapB^Strep^ were obtained on a UV-visible microplate reader (Infinite M20 pro, TECAN, Switzerland). The redox potentials of *So*NapB^Strep^ were examined through cyclic voltammetry (CV) electrochemical tests using an electrochemistry workstation (CHI660D, Chenhua Co. Ltd., Shanghai, China) with three electrode systems at ambient temperature. A platinum electrode and a saturated calomel electrode (SCE) were used as counter and reference electrodes, respectively. The working electrode was a glassy carbon electrode (1 cm diameter). All CV measurements were carried out in PIPES buffer (30 mM, pH = 6.5) with a scan rate of 50 mV s^−1^ under anaerobic conditions. Redox potentials were calculated by fitting the data with an *n* = 1 Nernstian curve as described before^48^.

### In silico analysis of NapB proteins

To identify proteins orthologous to *S. oneidensios* NapB, BLASTp was used to screen the available sequence data for proteobacteria. The retrieved sequences for NapB were subjected to evolutionary analyses and used to generate phylogenetic trees by using the neighbor-joining method in MEGA7^71^. The evolutionary history was represented by the bootstrap consensus tree inferred from 1000 replicates using the Neighbor-Joining method. Branches corresponding to partitions reproduced in less than 50% bootstrap replicates are collapsed. The evolutionary distances were computed using the Poisson correction method and are in the units of the number of amino acid substitutions per site. The analysis involved 60 amino acid sequences. All positions containing gaps and missing data were eliminated^71^. Multiple sequence alignments were performed by using Clustal Omega^72^.

Three-dimensional structures of *S. oneidensis* NapB were predicted using Phyre^73^. Available 3D structure of NapB of *R. sphaeroides* (PDB, 1ogy) was chosen as the template for structure prediction on the basis of high primary sequence identity and similarity^44^. The predicted structures were compared and visualized with the software Pymol (DeLano Scientific LLC).

### Statistics and reproducibility

For most analyses, at least four independent experiments were performed, from which biological replicates were obtained. In data presented as the mean ± standard deviation (SD), each replicate represents a biological replicate (an independent sample), rather than repeated measurement of the same sample. Student’s *t*-test was performed for pairwise comparisons. A *P* value of less than 0.05 was statistically significant.

### Reporting summary

Further information on research design is available in the Nature Research Reporting Summary linked to this article.

## Supplementary information

Supplementary Information

Description of Additional Supplementary Files

Supplementary Data 1

Supplementary Data 2

Reporting Summary

## Data Availability

The authors declare that all the data supporting the findings of this study are available within the article and its Supplementary Information file and from the corresponding author upon reasonable request. Uncropped gels are provided as supplementary figures in the Supplementary Information file (Supplementary Figs. 12, 13). Source data for the graphs and charts in the figures are available in Supplementary Data 1–2.
