# Supplementary material for: NapB Restores cytochrome c biosynthesis in bacterial dsbD-deficient mutants
Source: Commun Biol. 2022 Jan 21;5:87. doi: 10.1038/s42003-022-03034-3 (PMC8782879; doi:10.1038/s42003-022-03034-3)
Supplement: Supplementary file 1 — Supplementary Information [file 42003_2022_3034_MOESM1_ESM.pdf]

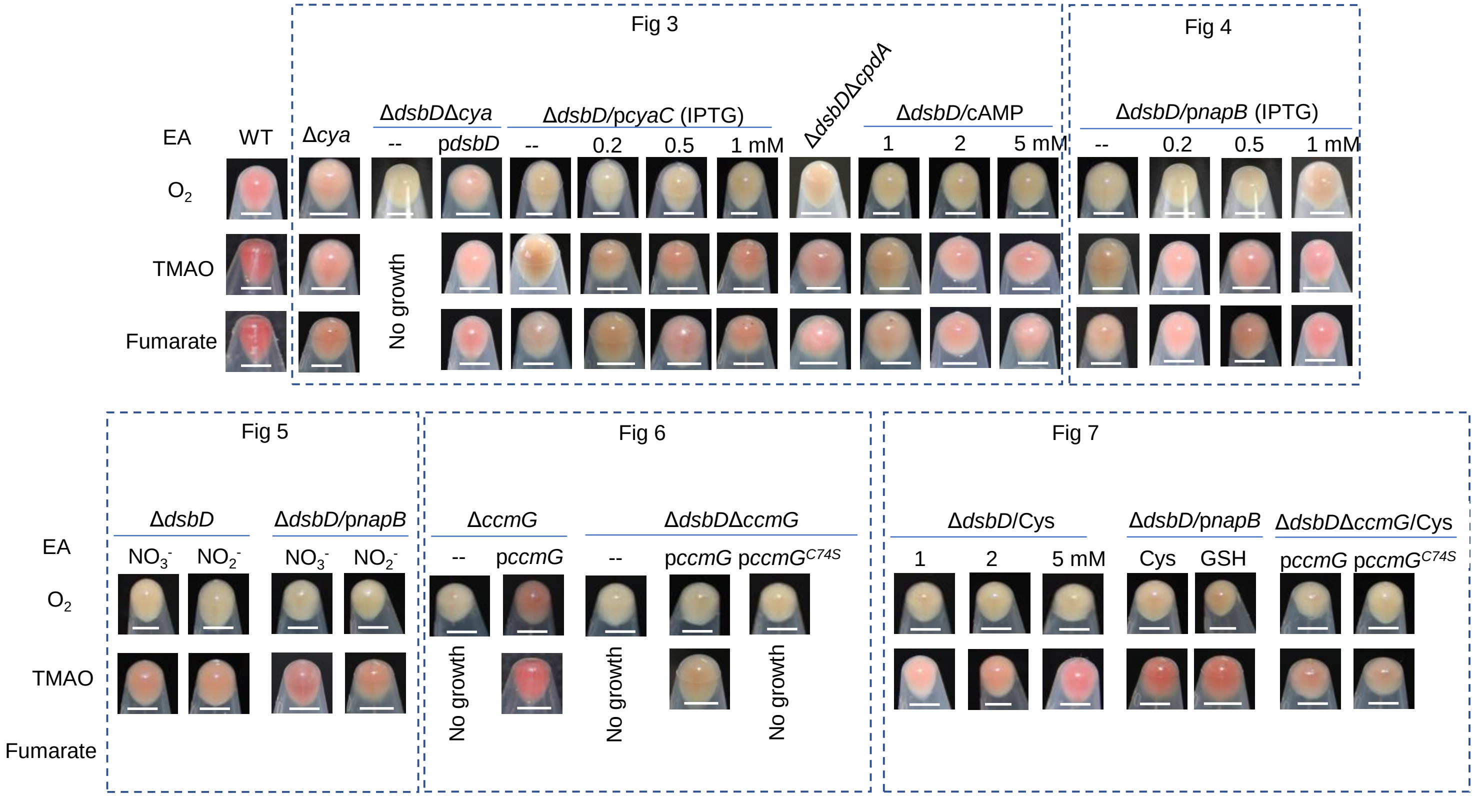

Supplementary Figure 1. The cell color phenotypes of indicated *S. oneidensis* strains grown under conditions specified. Shown were all strains used in this study supplemented to Figures presented in the main text. Scalebars in all subpanels, 5 mm. For supplemental materials to Fig. 5, the concentrations of NO_3^- and NO_2^- were 1 and 0.5 mM respectively. For supplemental materials to Fig. 7, the concentrations of Cys and GSH were 2 mM. All experiments were performed at least four times, and representative data were presented.

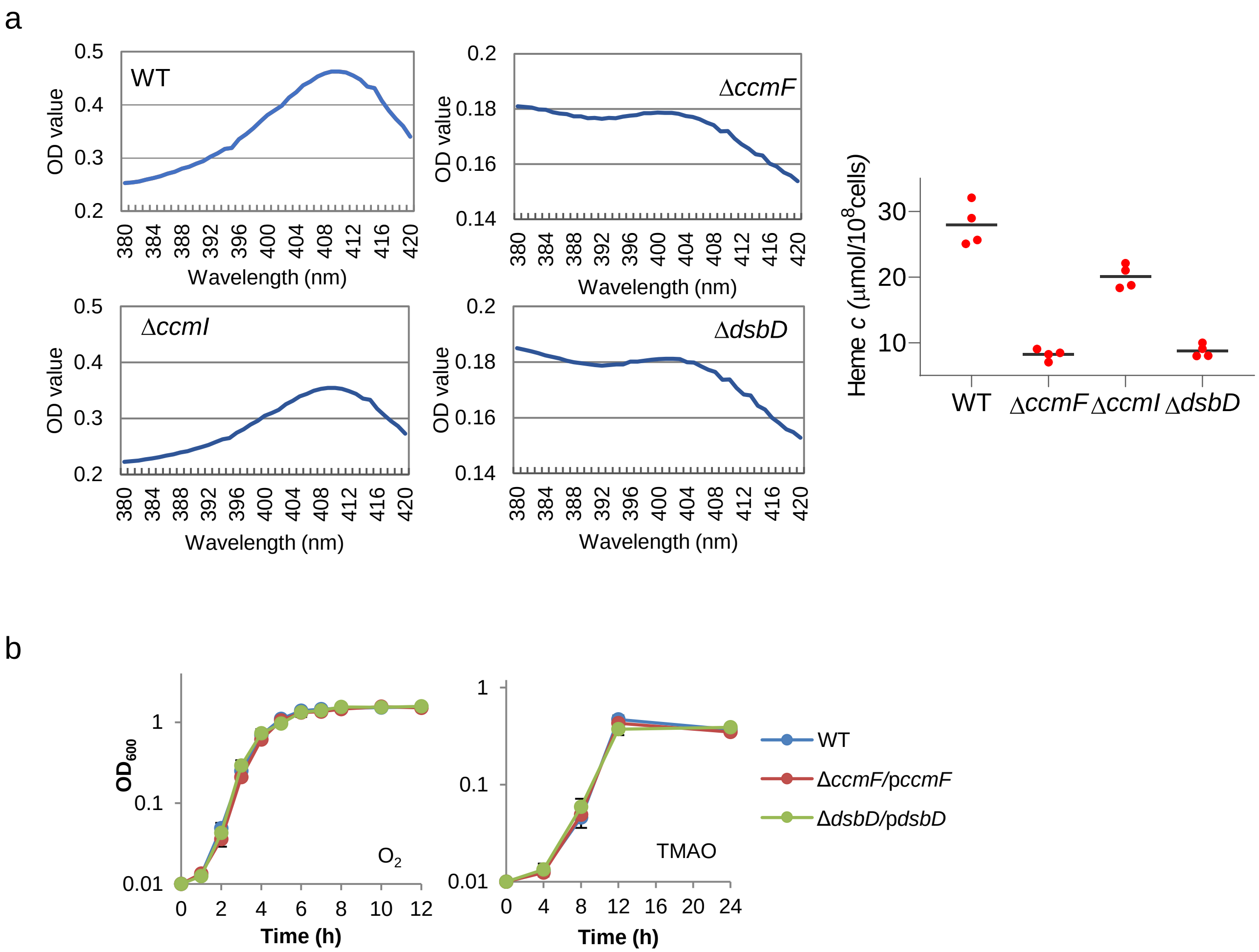

Supplementary Figure 2. Comparative analyses of Δ*dsbD* and Δ*ccmF*. **a** Quantification of heme *c* concentrations of Δ*dsbD* and Δ*ccmF* cells. Cells of the early stationary phase grown aerobically were collected and assayed for heme *c* levels with QuantiChrom^{TM} heme assay kit. The absolute values of heme *c* were calculated and shown. **b** Growth data of complementary strains for all experiments were performed at least three times, and the data were presented as the average ± SD (error bar).

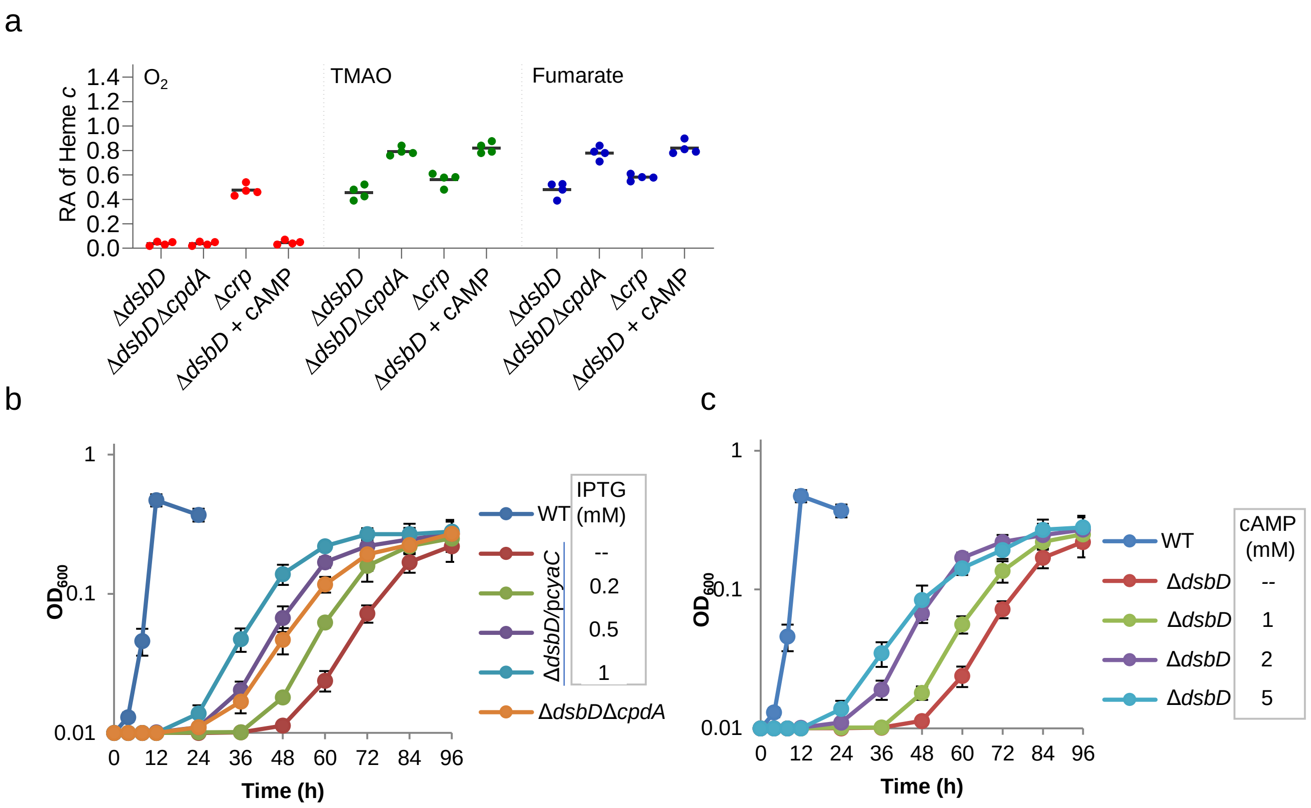

Supplementary Figure 3. cAMP is involved in anaerobic growth of the *S. oneidensis dsbD* mutant. **a** Heme *c* levels in strains grown to the early stationary phase. **b** Growth of indicated strains on TMAO. Expression of *cyaC* (p*cyaC*) was driven by IPTG-inducible promoter P*tac*. **c** Growth of Δ*dsbD* on TMAO supplemented with different concentrations of cAMP. All experiments were performed at least four times, and all data or average ± SD (error bar) were presented.

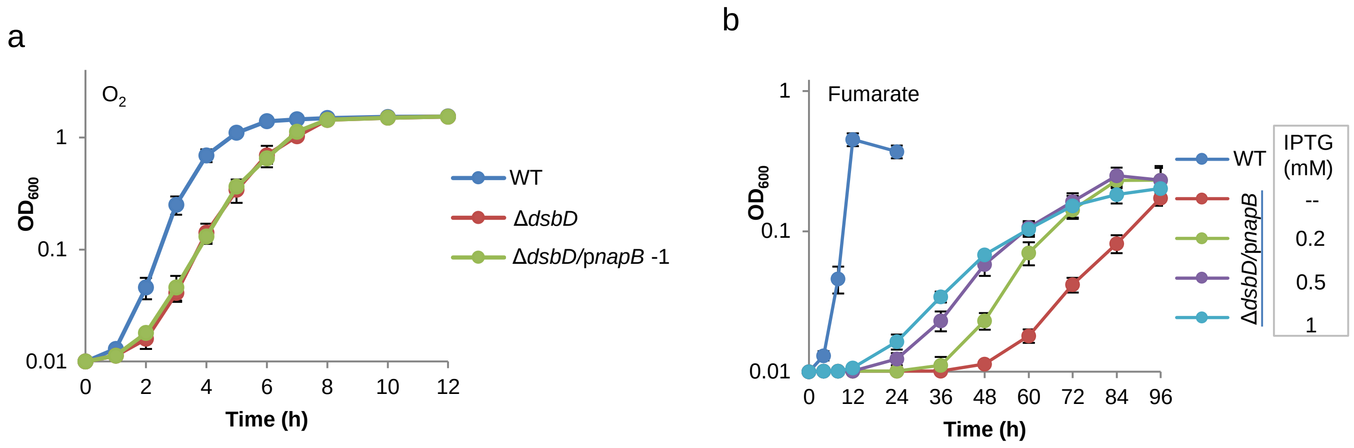

Supplementary Figure 4. Impacts of NapB on growth of *S. oneidensis dsbD* mutants on O_2 **(a)** and fumarate **(b)**. For aerobic growth, only data from 1 mM IPTG were shown. All experiments were performed at least four times, and all data or average ± SD (error bar) were presented.

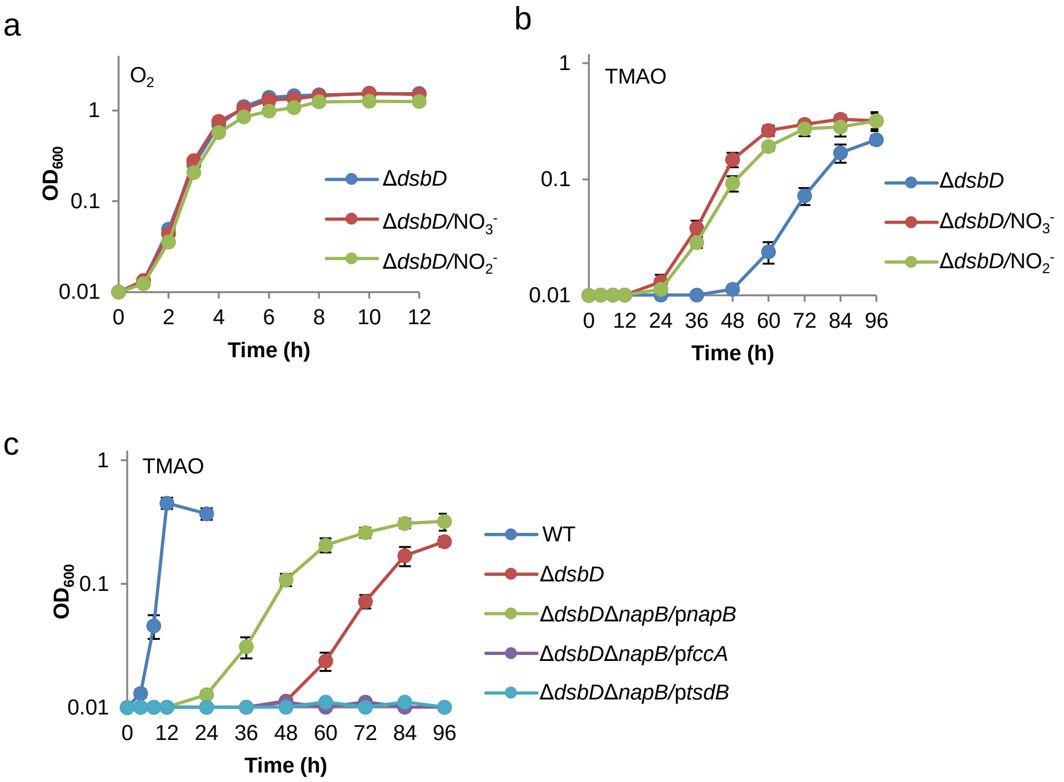

Supplementary Figure 5. Growth of *S. oneidensis* strains on O_2 and TMAO. **a** Growth of Δ*dsbD* on O_2 in the presence of nitrate or nitrite. **b** Growth of Δ*dsbD* on TMAO in the presence of nitrate or nitrite. **c** Growth of Δ*dsbD*Δ*napB* on TMAO overexpressing *napB*, *fccA*, or *tsdB* with 1 mM IPTG. The concentrations of NO_3^- and NO_2^- were 1 and 0.5 mM respectively. All experiments were performed at least four times, and the data were presented as the average ± SD (error bar).

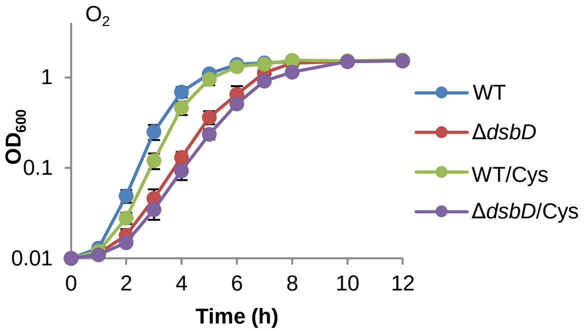

Supplementary Figure 6. Impacts of cysteine on growth of *S. oneidensis dsbD* mutants on O_2. The concentration of Cys was 5 mM. Cys at 2 mM or below did not affect growth. All experiments were performed at least four times, and the data were presented as the average ± SD (error bar).

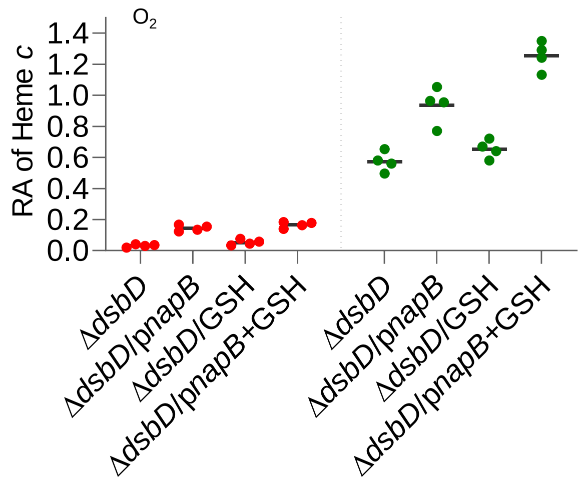

Supplementary Figure 7. Small reducing agents differ from NapB in modulating cyt *c* biosynthesis in the *dsbD* mutant. Heme *c* levels in indicated strains grown to the early stationary phase with or without 5 mM GSH. NapB expression was achieved in the presence of 1 mM IPTG. All experiments were performed at least four times, and all data or average ± SD (error bar) were presented.

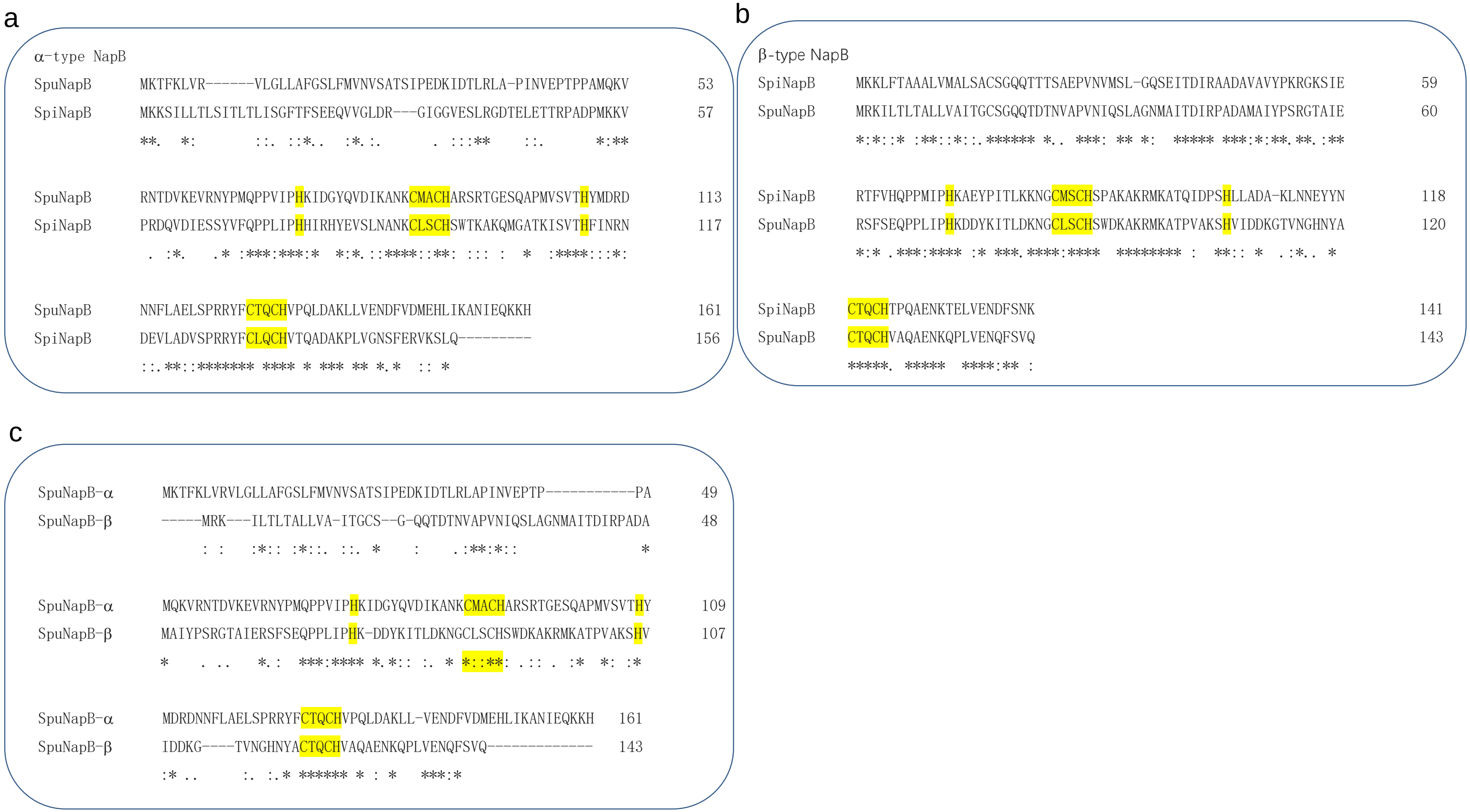

Supplementary Figure 8. Sequence alignment of NapB proteins from *S. putreficiens* and *S. piezotolerans*. HBM (CX_2CH) and two conserved His residues for heme attachment are highlighted. Clearly, the sequence identity between α-type NapBs (panel a, ~45%) is much lower than that between β-type NapBs (panel b, ~60%). The sequence identity between α-type and β-type NapBs of *S. putreficiens* is ~30% (panel c).

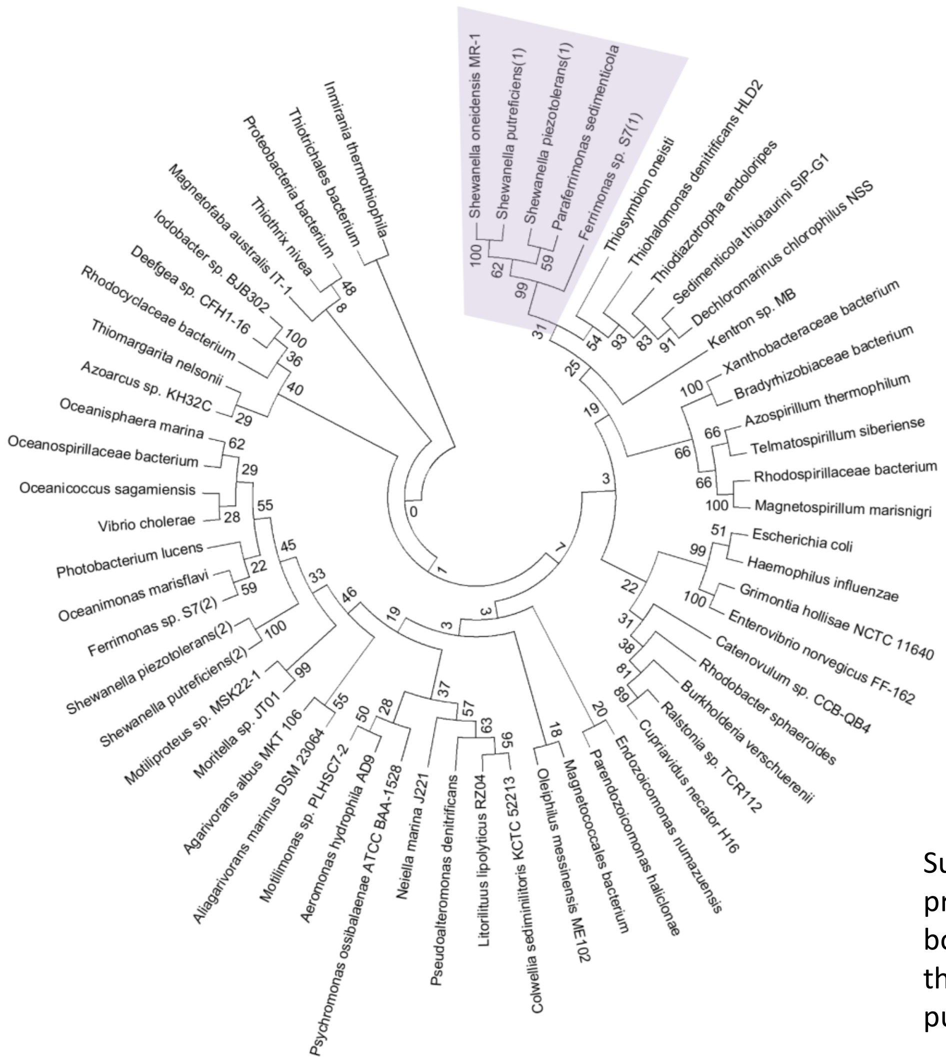

Supplementary Figure 9. Phylogenetic analysis of NapB proteins. The evolutionary history was represented by the bootstrap consensus tree inferred from 1000 replicates using the Neighbor-Joining method. β-type NapB proteins are in purple shadow.

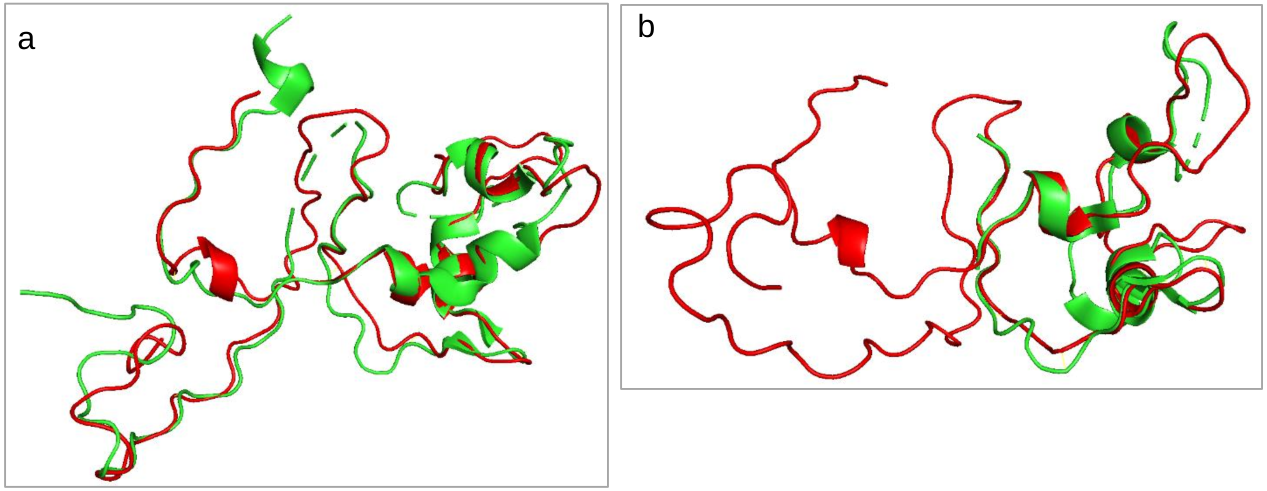

Supplementary Figure 10. Structural comparison of β-type NapB of *S. oneidensis* (red) and NapB proteins whose structure is resolved. **a** With α-type NapB of *Cupriavidus necator* (PDB accession number 3o5a, green). **b** With α-type NapB of *Haemophilus influenzae* (PDB accession number 1jni, green).

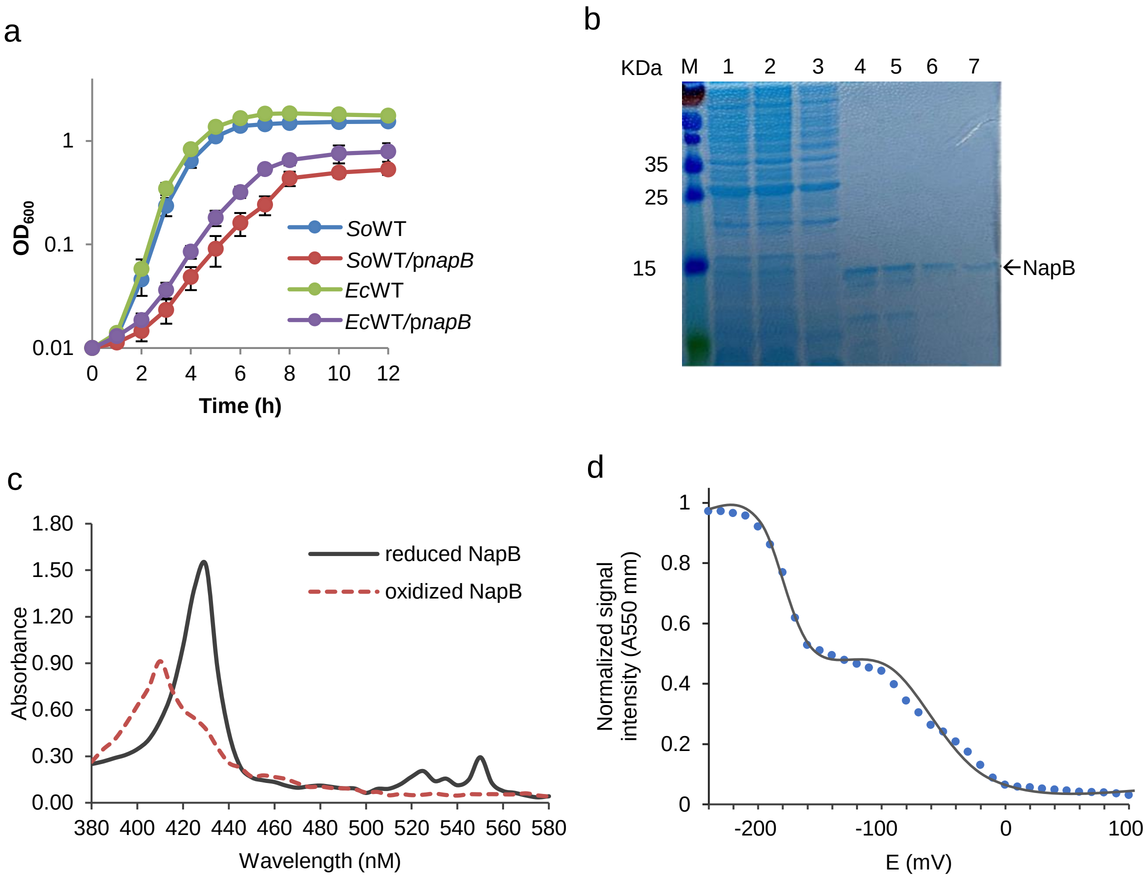

Supplementary Figure 11. NapB purification and biochemical characterization. **a** Impacts of NapB expression with 1 mM IPTG on aerobic growth of *S. oneidensis* and *E. coli* wild-type strains. The experiments were performed at least four times, and all data or average ± SD (error bar) were presented. **b** SDS-PAGE analysis of purified NapB proteins. Line: 1, total protein in the periplasm; 2, flow through; 3, wash out; 4-7, collected samples at UV peaks. **c** UV-visible absorption spectrum of NapB (100 μM) in 50 mM Tris-HCl, pH 8.0. **d** Plot of normalized signal intensity (measured from the intensity of the peak at 552 nm feature) versus potential. The data were fitted with an n = 1 Nernstian curve with Em = -30 mV and -180 mV for the two heme species. Conditions of measurement were as follows: NapB (0.2 μM) in 50mM Tris-HCl buffer, pH 8.0.

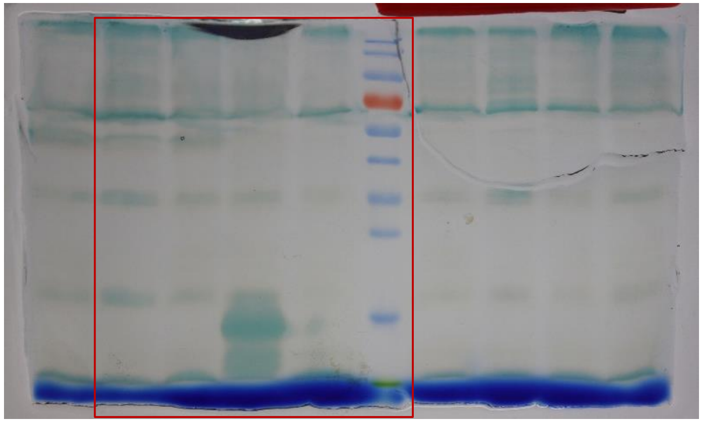

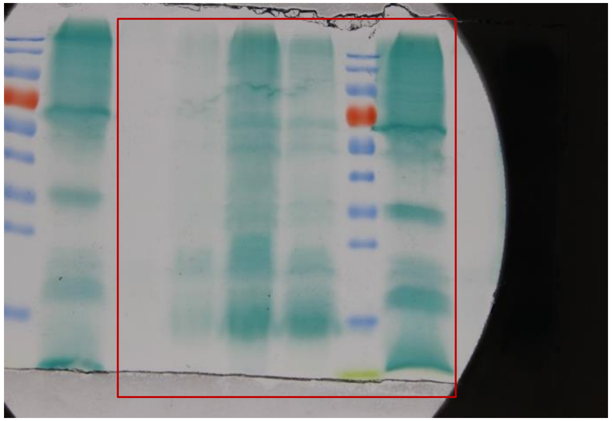

Supplementary Figure 12. Uncropped and unedited gel images for figure 4d. The parts in the red boxes were used to show the results.

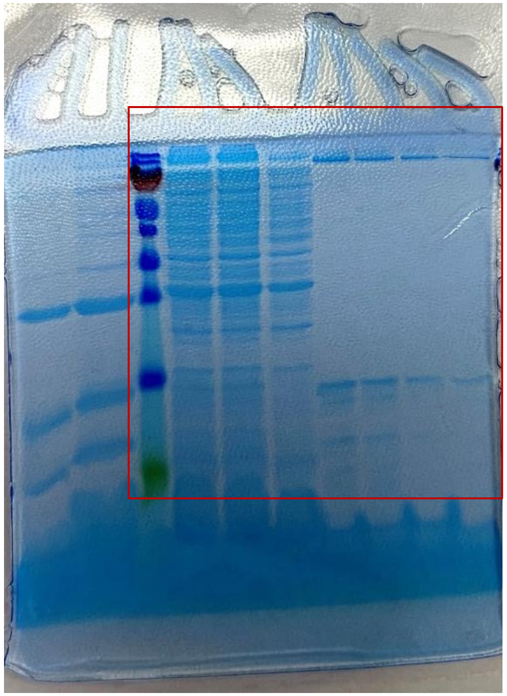

Supplementary Figure 13. Uncropped and unedited gel images for Supplementary Figure 11b. The part in the red box was used to show the results.

TABLE 1 Primers used in this study

Primers	Primer sequences
**In-frame deletion**	
HG0267-M5O	GGGGACAAGTTTGTACAAAAAAGCAGGCTGGAATTTGTTGGGCGCTTTA
HG0267-M5I	GGTCCGGGTTCGCTATCTATTCCTTCCAGAGCTGAATCCAG
HG0267-M3I	ATAGATAGCGAACCCGGACCCTACAAGCTGAAGCGGCGA
HG0267-M3O	GGGGACCACTTTGTACAAGAAAGCTGGGTGCGTAGACAGGGAAAACTCT
HG0696-M5O	GGGGACAAGTTTGTACAAAAAAGCAGGCTGCGAGTTTTTGTTGCACTTG
HG0696-M5I	GGTCCGGGTTCGCTATCTATTAATGCAGTGAAGGTAAGGCT
HG0696-M3I	ATAGATAGCGAACCCGGACCGTTTAACGAGCAAGGGGAAC
HG0696-M3O	GGGGACCACTTTGTACAAGAAAGCTGGGTTCAATCCCAAGACTGGTGAG
**Controlled expression**	
CcmF-CEF	CCGGAATTCATGATCCCAGAACTTGGACAC
CcmF-CER	CCGCTCGAGCCAATTATTGAGCGGTAGCT
NapB-CEF	CCGGAATTCATGAAAAAAATACTCACCTTAGCCGC
NapB-CER	CCCAAGCTTGGTTTATTGAGTTGAGAATTTA
CyaC-CEF	CGCGGATCCATGGCGGCATTCGTGTTTTTTCGATATGCCC
CyaC-CER	CCGCTCGAGGAGTCTTATAAAGGGGAAACACCGAACA
CcmG-CEF	CCGGAATTCATGAAGTCTCTCGCATTAATGGC
CcmG-CER	CGCGGATCCTTAATTCAGCTTTTTTGCGC
NapBspα-CEF	CCGGAATTCATGAAAACATTCAAATTAGTCAGAGTGTTAGG
NapBspα-CER	CCGCTCGAGCTAGTGTTTTTTCTGCTCAATATTGGC
NapBspβ-CEF	CCGGAATTCATGAAAAAAATACTCACCTTAGCTGCC
NapBspβ-CER	CCGCTCGAGCTATTGGTTTGAGAACTTATTCTCGAC

***lacZ* reporters**	
P*nap*-LRF	CCGGAATTCCCACACAGGCATCCCACT
P*nap*-LRR	CGCGGATCCGTAATCATGGTCATTTCTTGTTCTAGGGGCTCGAC
**Protein expression**	
NapB-PEF	CCGGAATTCATGAAAAAAATACTCACCTTAGCCGC
NapB-PER	CCCAAGCTTGGTTTATTGAGTTGAGAATTTA
Ccmec-PEF	CCGGAATTCGTGGGTATGCTTGAAGCCAGAGAGTTAC
Ccmec-PER	CCCAAGCTTTTATTTACTCTCCTGCGGCGACAAATGTTGCATCGC
