# Supplementary material for: NapB Restores cytochrome c biosynthesis in bacterial dsbD-deficient mutants
Source: Commun Biol. 2022 Jan 21;5:87. doi: 10.1038/s42003-022-03034-3 (PMC8782879; doi:10.1038/s42003-022-03034-3)
Supplement: Supplementary file 2 — Description of Additional Supplementary Files [file 42003_2022_3034_MOESM2_ESM.pdf]

Description of Additional Supplementary Files

File name: Supplementary Data 1
Description: Source data for the graphs and charts.

File name: Supplementary Data
Description: Source data for the graphs and charts.
